# Real-world Bleeding Outcomes and Costs Following Vascular Graft Anastomosis Using PROLENE Sutures with HEMO-SEAL Technology in Patients Undergoing Abdominal Aortic Aneurysm Repair

**DOI:** 10.36469/9806

**Published:** 2017-09-01

**Authors:** Nadia Y. Sutton, Niels-Derrek Schmitz

**Affiliations:** 1 Ethicon, Somerville, NJ, USA; 2 Ethicon, Norderstedt, Germany

**Keywords:** abdominal aortic aneurysm (aaa), real-world outcomes, database, anastomosis, suture, prolene, hemo-seal

## Abstract

**Background:** Suture hole bleeding is a common complication of vascular graft anastomosis that has potential to prolong vascular procedures, increase costs, and compromise patient outcomes.

**Objectives:** Compare real-world bleeding-related outcomes and costs following vascular anastomosis using PROLENE sutures with HEMO-SEAL technology (HEMO-SEAL sutures) compared with standard PROLENE sutures in patients receiving abdominal aortic aneurysm (AAA) repair in the United States.

**Methods:** AAA repair procedures using hemostats and either HEMO-SEAL sutures or standard PROLENE sutures were identified from 2009 to 2013 using the Premier Healthcare Database. The primary outcome was the number and cost of hemostat units. Secondary outcomes were number and cost of sutures, bleeding complications, and transfusions.

**Results:** A total of 5082 discharges for AAA repairs using hemostats and HEMO-SEAL sutures or standard PROLENE sutures were identified. HEMO-SEAL sutures were used in 79 (1.6%) discharges, standard PROLENE sutures were used in 4946 (97.3%); both sutures (excluded from the analysis) were used in 57 (1.1%). Discharge demographics were similar across suture groups, with the exception of disease severity; the HEMO-SEAL suture group had a higher proportion of minor discharges and a lower proportion of extreme discharges compared with the standard PROLENE suture group. Mean number of hemostat units used per discharge (2.34 vs 3.30; median = 2.0 in both groups; p=0.026) and median hemostat costs per discharge ($111 vs $186; p<0.01) were significantly lower in the HEMO-SEAL suture group compared with the standard PROLENE suture group. Fewer sutures per discharge (p<0.0001), lower mean costs of sutures per discharge, higher median costs of sutures per discharge (p=0.0045), and fewer transfusions (0.0019) were also seen in the HEMO-SEAL suture group compared with the standard PROLENE suture group. No statistically significant difference in bleeding complications was observed between suture groups.

**Conclusion:** The results indicate that real-world use of HEMO-SEAL sutures may be associated with reduced hemostat usage and costs, and reduced bleeding that requires additional hemostats and/or transfusions.

## BACKGROUND

Needle hole bleeding (NHB) is a common complication of vessel anastomosis that occurs when even ideal suturing techniques are used and has the potential to increase intra-operative blood loss, lengthen operating time, compromise patient outcomes, and increase costs.[1-4] Patients at particular risk for NHB include those with hypertension, bleeding disorders, chronic liver disease, and renal failure, and those requiring anticoagulants or platelet inhibitors for the prevention of intra-operative thrombus formation.[1,2,4,5] Use of conventional synthetic grafts such as polytetrafluoroethylene [PTFE] grafts further exacerbate NHB.[3,5-8]

Early research demonstrated that using sutures with a small needle-to-suture (N:S) diameter ratio (eg, 1:1) reduces the discrepancy between the diameter of the needle hole and the diameter of the suture filling it, thereby significantly reducing NHB.[8,9] The ROLENE suture with HEMO-SEAL technology (HEMOSEAL suture) represents the latest breakthrough in suture technology. HEMO-SEAL technology tapers down the PROLENE suture at the needle attachment site which is seamlessly compressed into the end of an eyeless 'swaged' needle during production. The technology reduces the needle swage area, allowing for finer diameter needles to be aligned with the tapered suture and providing a further decreased N:S diameter ratio.[10] In a pre-clinical study comparing the NHB rate of an end-to-end synthetic expanded PTFE (ePTFE) to synthetic ePTFE vascular graft anastomosis sutured using the HEMO-SEAL suture (C-1 needle) with the NHB rate of a standard 5.0 PROLENE polypropylene suture (C-1 needle), the HEMO-SEAL suture resulted in a 67% reduction in mean NHB rate at graft anastomosis compared to the standard PROLENE suture (NHB rate of 0.63 ±0.13 ml/min for the HEMO-SEAL PROLENE suture compared to 1.94 ±0.23 ml/min for the standard PROLENE suture).[11]

The objective of this study was to compare real-world bleeding-related outcomes and costs following vascular graft anastomosis with HEMO-SEAL sutures compared to standard PROLENE sutures in patients undergoing abdominal aortic aneurysm (AAA) repair in the United States. Standard PROLENE sutures were selected for this study due to the appropriateness of their use in AAA procedures, and to provide a truer perspective of the impact of the HEMO-SEAL technology.

## METHODS

### Study Design and Data Source

This retrospective study used data from the Health Insurance Portability and Accountability Act (HIPAA) compliant Premier Healthcare database. The database contains de-identified inpatient data for over 75 million discharges (6 million discharges per year since 2010) from more than 670 hospitals across the US.[12] Of particular importance to this study, the database contains a date-stamped log of all billed items, including procedures, laboratory, and diagnostic services, for each discharge.

The study included discharges for patients of all ages between 2009 and 2013 with a primary procedure of AAA or possible AAA, according to the International Classification of Diseases, Ninth Edition, Procedure Coding System (ICD-9-PCS) codes (Table 1).[13] Coronary artery bypass graft (CABG), cardiac valve repair and replacement, aortic root or open thoracic aortic aneurysm, and total repair of truncus arteriosus procedures were also considered, however, small numbers of discharges reporting use of HEMO-SEAL sutures for these procedures precluded their inclusion in the analysis.

For all AAA or possible AAA discharges, hospital charge codes were examined to determine hemostat and

HEMO-SEAL suture or standard PROLENE suture use. A total of 31 349 unique hospital charge codes were used to identify hemostat use. The terms 'HEMO-SEAL', 'HS', 'HEMOSEAL', 'PROLENE', as well as the product identification codes for Ethicon HEMO-SEAL suture products were used to identify potential HEMO-SEAL suture hospital charge codes. A total of 185 unique hospital codes were identified; 71 (38.4%) of these codes specifically included the terms 'HEMO-SEAL', 'HS', or 'HEMOSEAL' in conjunction with other identifiable terms (eg, 'Ethicon') and product identification codes and were characterized as 'definitely HEMO-SEAL', 110 (59.5%) included other identifiable terms in conjunction with product identification codes and were characterized as 'most likely HEMO-SEAL', and 4 (2.2%) included other identifiable terms and product identification codes but provided insufficient additional information to say the suture was most likely HEMO-SEAL and were characterized as 'possibly HEMO-SEAL' (Supplemental Table 1). A total of 993 unique hospital charge codes were identified as being standard PROLENE sutures (eg, non-HEMOSEAL sutures). Discharges reporting no use of hemostats, HEMO-SEAL sutures, or standard PROLENE sutures were excluded.

Outcomes of interest in the study were resource use, costs, and clinical outcomes related to bleeding. The primary outcome was the number and cost of hemostat units used. Secondary outcomes were number and cost of sutures, bleeding complications, and transfusions. The cost of hemostats and sutures were taken directly from the Premier database and represent the actual cost to treat the patient, including variable (direct) costs and fixed (overhead) costs. Discharges that included bleeding complications and/or transfusions were identified using Current Procedural Terminology (CPT) codes, diagnosis codes, procedure codes, hospital charge codes, and Healthcare Common Procedure Coding System (HCPCS) codes.

Table 1: ICD-9 PCS Discharge Codes Used to Identify Procedures for a Diagnosis of AAA or Possible AAA

| ICD-9 PCS Code | Long Description of Procedure |
|---|---|
| 38.34 | Resection of vessel with anastomosis, aorta |
| 38.36 | Resection of vessel with anastomosis, abdominal arteries |
| 38.37 | Resection of vessel with anastomosis, abdominal veins |
| 38.44 | Resection of vessel with replacement, aorta, abdominal |
| 38.64 | Other excision of vessels, aorta, abdominal |
| 39.51 | Clipping of aneurysm |
| 39.52 | Other repair of aneurysm |
| 39.54 | Re-entry operation (aorta) |
| 39.59 | Other repair of vessel |
| 39.71 | Endovascular implantation of other graft in abdominal aorta |

AAA: abdominal aortic aneurysm
Source: Centers for Medicare & Medicaid Services[13]

## STATISTICAL ANALYSIS

Demographic characteristics between groups were compared by two-sided chi-squared test. Number of hemostat units, number of sutures, and cost outcomes between groups were compared using Wilcoxon rank sum test, to account for non-normality of this data. These statistical methods were utilized vs propensity score matching because there was an asymmetric identification of HEMO-SEAL sutures vs PROLENE sutures in the sample. HEMO-SEAL sutures are a newer technology with less widespread use which limited our ability to match sample sizes between the groups.

Bleeding complications and transfusions between groups were compared using the Fisher's Exact test. A p-value <0.05 was considered statistically significant. All statistical analyses were conducted using R (version 3.2.2).

## RESULTS

### Patient and Hospital Characteristics

A total of 5025 discharges for AAA or possible AAA with hemostat use and use of HEMO-SEAL sutures or standard PROLENE sutures were eligible for analysis. A total of 79 discharges comprised the HEMOSEAL suture group while the remainder (4946) comprised the standard PROLENE suture group (Figure 1). Discharges reporting use of both suture types were excluded from the analysis.

**Figure 1.** Flow Chart of the Study Sample Selection Process

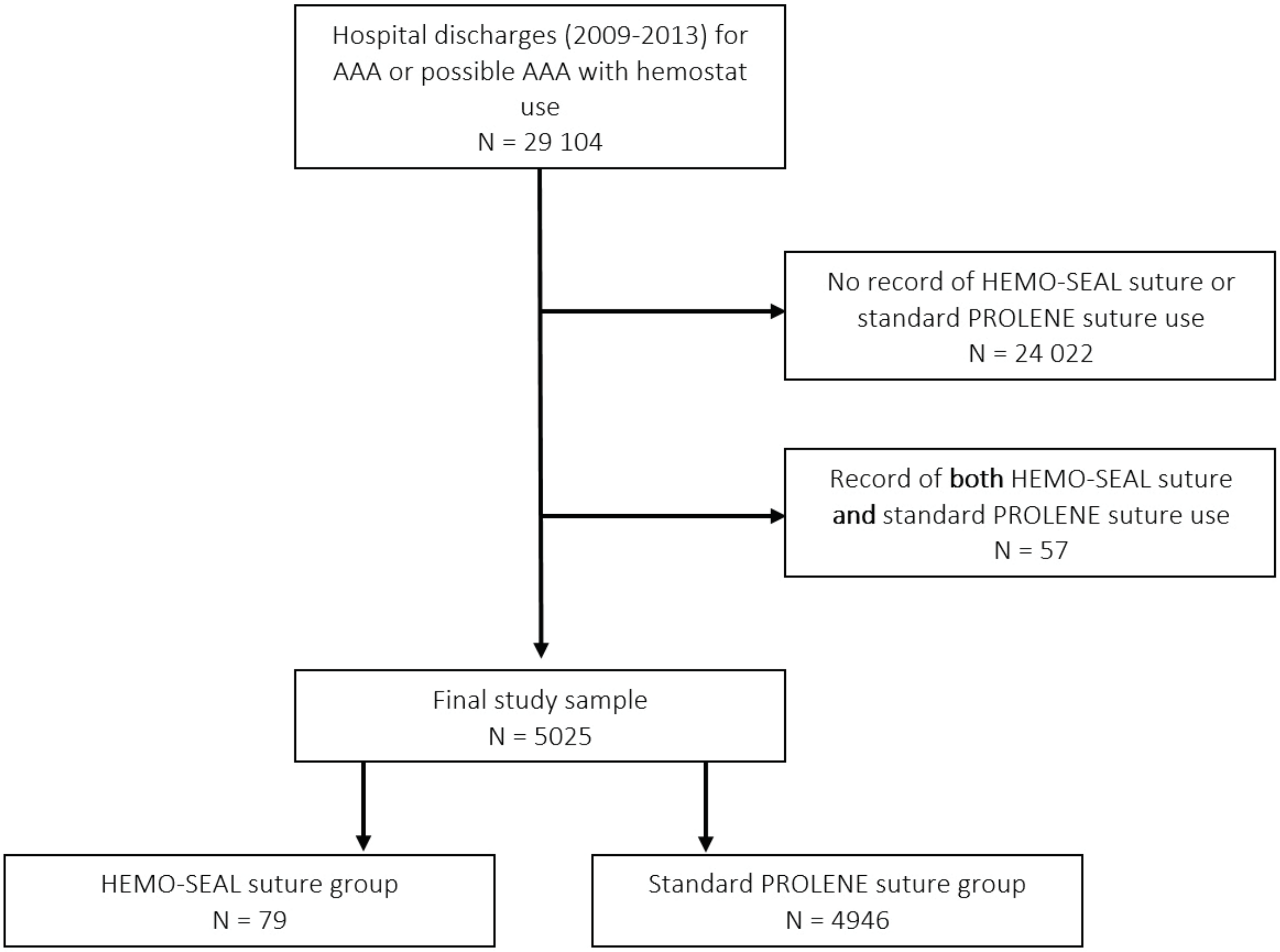

AAA: abdominal aortic aneurysm

Patient demographics for discharges (ie, age, sex, payer type) were similar across suture groups. The majority of discharges (41.8%) were for patients aged between 65 and 74 years in the HEMO-SEAL suture group with a larger number of patients aged over 74 years in the standard PROLENE suture group (37.9%) (p = 0.705) (Table 2). There were significantly fewer female discharges in both suture groups and Medicare was the most frequent payer for both suture groups.

**Table 2.** Summary of Patient Demographic and Hospital Characteristics by Suture Group

| | HEMO-SEAL Suture Group N = 79 n (%) | Standard PROLENE Suture Group N = 4946 n (%) | p-value* |
|---|---|---|---|
| **Age (years)** | | | |
| Under 45 | 2 (2.5) | 179 (3.6) | |
| 45 to 54 | 4 (5.0) | 266 (5.4) | |
| 55 to 64 | 10 (12.7) | 867 (17.5) | 0.705 |
| 65 to 74 | 33 (41.8) | 1759 (35.6) | |
| Over 74 | 30 (38.0) | 1875 (37.9) | |
| **Sex** | | | |
| Female | 21 (26.6) | 1284 (26.0) | 0.901 |
| Male | 58 (73.4) | 3662 (74.0) | |
| **Payer** | | | |
| Medicare | 62 (78.5) | 3452 (69.8) | |
| Medicaid | 2 (2.5) | 168 (3.4) | |
| Cash | 1 (1.3) | 106 (2.1) | 0.236 |
| Commercial | 14 (17.7) | 961 (19.4) | |
| Other | 0 | 259 (5.2) | |
| **Severity of illness, n (%)** | | | |
| Minor | 24 (30.4) | 929 (18.8) | |
| Moderate | 29 (36.7) | 1655 (33.5) | |
| Major | 19 (24.1) | 1257 (25.4) | 0.015 |
| Extreme | 7 (8.9) | 1060 (21.4) | |
| Unknown | 0 | 45 (0.9) | |
| **Hospital provider area** | | | |
| Midwest | 1 (1.3) | 576 (11.6) | |
| Northeast | 3 (3.8) | 411 (8.3) | |
| South | 67 (84.8) | 2853 (57.7) | <0.0001 |
| West | 8 (10.1) | 1074 (21.7) | |
| Not known | 0 | 32 (0.6) | |
| **Hospital location** | | | |
| Rural | 47 (59.5) | 525 (10.1) | |
| Urban | 32 (40.5) | 4389 (88.7) | <0.0001 |
| Not known | 0 | 32 (0.6) | |
| **Hospital affiliation** | | | |
| Non-teaching | 76 (96.2) | 3006 (60.8) | |
| Teaching | 3 (3.8) | 1908 (38.6) | <0.0001 |
| Not known | 0 | 32 (0.6) | |
| **Hospital number of beds** | | | |
| Under 200 | 3 (3.8) | 221 (4.5) | |
| 200 to 400 | 55 (69.6) | 1751 (35.4) | <0.0001 |
| Over 400 | 21 (26.6) | 2974 (60.1) | |

*p-values calculated using the 2-sided chi-squared test; a p-value <0.05 was considered statistically significant.

Classification of illness severity was performed using the 3M™ All Patient Refined Diagnosis-Related Group (APR DRG) Classification System.[14] The 3M APR DRG Classification System assigns a severity of illness subclass using a sophisticated clinical logic that evaluates patient discharge information such as principal diagnosis, secondary diagnoses, age, sex, operating-room (OR) procedures, and non-OR procedures. The severity of illness subclasses are minor, moderate, major, and extreme; patients with higher severity of illness have multiple co-morbid conditions involving multiple organ systems and tend to have poor outcomes. The 3M APR DRGs reflect the complete cross-section of patients seen in an acute care setting and are used widely throughout the US for payment or public quality reporting purposes. The HEMO-SEAL suture group tended to have a higher proportion of minor discharges compared with the standard PROLENE group (30% vs. 19%) and a lower proportion of extreme discharges (9% vs 21%). The proportion of discharges that were either moderate or major was similar across suture groups. Overall, the difference between suture groups in severity of illness level was significant at p = 0.015.

Most discharges were reported from non-teaching hospitals (96.2% for the HEMO-SEAL suture group and 60.8% for the standard PROLENE group; p < 0.0001) with mid-sized hospitals (ie, 200 to 400 beds) reporting more use of HEMO-SEAL sutures (69.6%) and large-sized hospitals (ie, over 400 beds) reporting more use of standard PROLENE sutures (60.1%) (p < 0.0001). A higher percentage of southern (84.8% compared to 57.7%) and rural (p < 0.0001) hospitals in the US favored HEMO-SEAL sutures) (Table 2).

## Hemostat Use and Costs

Use of HEMO-SEAL sutures compared with standard PROLENE sutures was associated with significantly fewer hemostat units and lower hemostat costs per discharge (Figure 2). The HEMO-SEAL suture group used an average of 2.34 hemostat units per discharge (median = 2.0), compared with 3.30 for the standard PROLENE suture group (median = 2.0) (p = 0.026 based on Wilcoxon rank sum test). The mean cost of hemostat units per discharge was $308 for the HEMO-SEAL suture group compared with $621 for the standard PROLENE group (median cost of $111 vs $186; p = 0.0034 based on Wilcoxon rank sum test).

The types of hemostats used were similar across suture groups, apart from increased use of oxidized regenerated cellulose (ORC) in the standard PROLENE group (data not shown).

**Figure 2.** Number and Cost of Hemostat Units per Discharge, by Suture Group

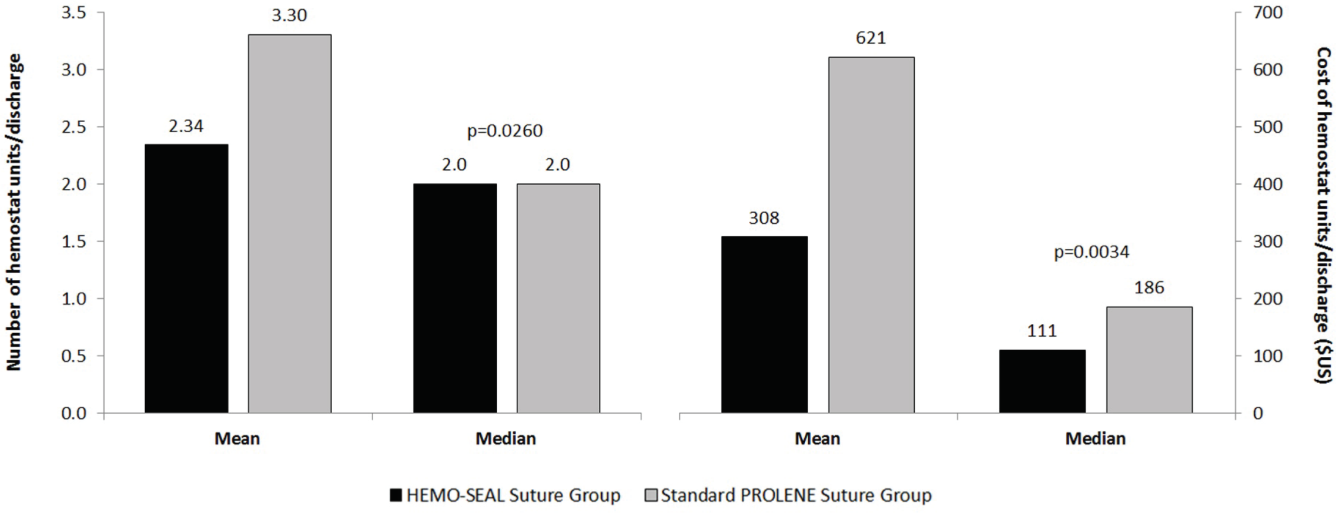

P-values calculated using two-sided Wilcoxon rank sum test and compared against the p<0.05 significance level.

### Secondary Outcomes

Results of the secondary analyses are summarized in Table 3. The HEMO-SEAL suture group used an average of 2.86 sutures per discharge (median = 3.0), compared with 6.74 for the standard PROLENE suture group (median = 4.0) ($p < 0.0001$ based on Wilcoxon rank sum test. The mean cost of sutures per discharge was lower for the HEMO-SEAL suture group compared with the standard PROLENE suture group ($64 vs $76) but the median cost of sutures was higher for the HEMO-SEAL suture group compared with the standard PROLENE suture group ($50 vs $36; p = 0.0045 based on Wilcoxon rank sum test).

The vast majority of all discharges (98.9%) reported bleeding complications; the proportion of discharges reporting bleeding complications was similar across suture groups (p = 0.2139). However, a significantly smaller proportion of discharges in the HEMO-SEAL suture group compared with the standard PROLENE suture group reported transfusions (27% vs 44%, p = 0.0019).

**Table 3.** Summary of the Results for the Secondary Outcomes

| | HEMO-SEAL Suture Group N = 79 | Standard PROLENE Suture Group N = 4946 | p-value* |
|---|---|---|---|
| **Number of sutures** | | | |
| Mean (SD) | 2.86 (1.82) | 6.74 (7.57) | |
| Median (Q1, Q4) | 3.0 (1, 4) | 4.0 (2, 8) | <0.0001 |
| **Cost of sutures ($US)** | | | |
| Mean (SD) | 64.46 (48.61) | 75.70 (156.87) | |
| Median (Q1, Q4) | 50 (37, 89) | 36 (17, 79) | 0.0045 |
| Bleeding complications, n (%) | 77 (97.4) 4 | 893 (98.9) | 0.2139 |
| Transfusions, n (%) | 21 (26.6) | 2184 (44.2) | 0.0019 |

Results are reported per discharge.
*Number and cost of sutures between groups were compared using Wilcoxon rank sum test, to account for nonnormality of this data; bleeding complications and transfusions between groups were compared using the Fisher's Exact test. All p-values were compared against the $p < 0.05$ significance level.

## DISCUSSION

This study is the first to characterize the real-world bleeding-related outcomes and costs following vascular anastomosis with HEMO-SEAL sutures compared to standard PROLENE sutures in patients undergoing a vascular procedure in the US.

The findings suggest that use of HEMO-SEAL sutures may be associated with significantly fewer hemostat units and lower hemostat costs per discharge compared with use of standard PROLENE sutures in patients undergoing an AAA or possible AAA procedure. Use of HEMO-SEAL sutures may also be associated with fewer transfusions per discharge compared with use of standard PROLENE sutures. HEMO-SEAL sutures were associated with fewer sutures per discharge and lower mean costs of sutures per discharge but higher median costs of sutures per discharge compared with the standard PROLENE sutures. Taken together, the results indicate that real-world use of HEMO-SEAL sutures may be associated with reduced hemostat usage and costs, and reduced bleeding that requires additional hemostats and/or transfusions. Reduced bleeding may also translate into shorter operating times, shorter hospital stays, and improved patient outcomes.[1,3,4,5,7,15,16,17]

Limitations of this retrospective data analysis warrant caution in the interpretation of the results.[18] Of primary concern is potential bias resulting from uncontrolled confounders, and so these results should be considered exploratory in nature. However, even if an adjustment controlling for confounders was considered, there is a potential of having sparse or missing data for potential confounders due to the sample size of the HEMO-SEAL suture group being smaller than the PROLENE suture group.

The analysis was limited to those data collected in the Premier database. Other patient characteristics and variables that may impact choice of suture, NHB, and study outcomes were not available from the Premier dataset and were not controlled for in the analysis. Such characteristics/variables include admission type (elective, urgent or emergent), co-morbidities, disease severity in terms of stable or ruptured aneurysm, procedure type (traditional open surgery or endovascular surgery), prosthetic graft material, number of anastomosis, intraoperative variables, and use and dosage of heparin.[19,20] Since suture groups were similar in terms of the demographic characteristics (age, sex, and payer) that were available, propensity score matching was not employed to improve the similarity of the analyzed groups. While the HEMO-SEAL suture group had a significantly higher proportion of minor discharges and a significantly lower proportion of extreme discharges, these results must be interpreted with caution. The assignment of a discharge to a severity of illness subclass takes into consideration the principal diagnosis, secondary diagnoses, age, the presence of certain OR and non-OR procedures, as well as the interaction of these factors.[14] A patient can have multiple procedures during their hospital stay, which may impact their severity of illness subclass. Without further information regarding secondary diagnoses and in-hospital procedures, it is impossible to distinguish the impact of each of these factors on the differences seen in the severity of illness subclass between the HEMO-SEAL suture group and the standard PROLENE suture group. It may be that the HEMO-SEAL suture group was healthier upon admission compared with the standard PROLENE suture group, or it may be that the HEMO-SEAL suture group received fewer OR and non-OR procedures during their stay, or it may be some combination of the two. Without further information regarding the secondary diagnoses, it is also difficult to determine if co-morbid conditions would have placed patients at a higher risk of the types of outcomes considered in this study (e.g., hemostat use, suture use, bleeding complications, and transfusions).

Another potential limitation relates to the identification of sutures. A total of 183 unique hospital codes were identified as being HEMO-SEAL sutures and a total of 993 unique hospital charge codes were identified as being standard PROLENE sutures. A high proportion of the codes for HEMO-SEAL sutures were characterized as being either 'definitely HEMO-SEAL' or 'most likely HEMO-SEAL', ensuring that all HEMO-SEAL sutures included in the analysis were indeed HEMO-SEAL sutures. Similarly, standard PROLENE sutures that were not explicitly non-HEMO-SEAL sutures were excluded from the analysis. The authors felt it was important for the integrity of the study to be conservative in this identification so as to minimize potential risk of including either non-HEMO-SEAL sutures in the HEMO-SEAL suture group or non-PROLENE sutures in the standard PROLENE suture group. Consequently, eligible discharges with HEMO-SEAL or standard PROLENE sutures may have been excluded from the analysis. The impact of excluding potentially eligible sutures cannot be known. Further research is required to confirm the findings of this analysis. In particular, prospective and/or retrospective studies controlling for potential confounders and risk of selection bias are needed.

## CONCLUSION

In conclusion, this study of real-world US data suggests that use of HEMO-SEAL sutures may be associated with reduced hemostat usage and costs, and reduced bleeding that requires additional hemostats and/or transfusions. Further prospective and/or retrospective studies are needed to confirm these findings.

## DECLARATIONS

### Availability of data and materials

The datasets analyzed during the current study are available from the corresponding author on reasonable request.

### Competing Interests

NYS and NDS are employees of Ethicon.

### Funding

The Premier analysis and manuscript were funded by Ethicon.

### Authors' Contributions

NYS and NDS conceived the study and were major contributors in designing the study and writing the manuscript. NYS and NDS read and approved the final manuscript.

### Acknowledgements

The analysis was conducted by Trinity Partners; Kevin Francis, Isabella Kredlow, Elnara Eynullayeva.

## Figures and Tables

**Figure:** Supplementary Content

